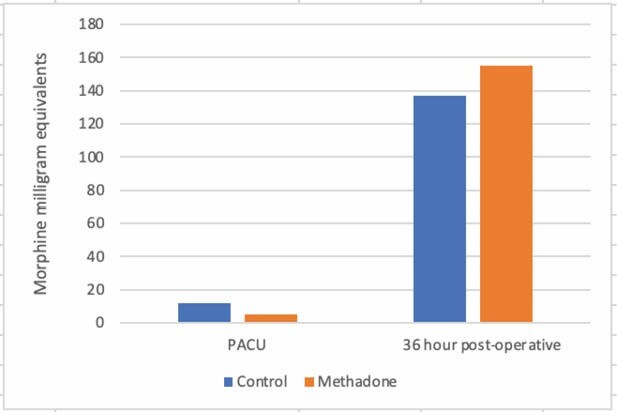# 768 Intraoperative Use of Intravenous Methadone and Post-Operative Opioid Requirements in Adult Burn Patients

**DOI:** 10.1093/jbcr/irad045.243

**Published:** 2023-05-15

**Authors:** Christopher LaChapelle, Jenny Ringqvist, David Pham, Vrinda Shukla, Caitlin O'Connor, Braden Wu, Aditee Ambardekar

**Affiliations:** University of Utah, Salt Lake City, Utah; University of Texas Southwestern Medical Center, Dallas, Texas; UT Southwestern Medical Center, Dallas, Texas; University of Texas Southwestern Medical Center, Dallas, Texas; UT Southwestern Medical Center, Dallas, Texas; The University of Texas Southwestern Medical School, Plano, Texas; UT Southwestern Medical Center, Dallas, Texas; University of Utah, Salt Lake City, Utah; University of Texas Southwestern Medical Center, Dallas, Texas; UT Southwestern Medical Center, Dallas, Texas; University of Texas Southwestern Medical Center, Dallas, Texas; UT Southwestern Medical Center, Dallas, Texas; The University of Texas Southwestern Medical School, Plano, Texas; UT Southwestern Medical Center, Dallas, Texas; University of Utah, Salt Lake City, Utah; University of Texas Southwestern Medical Center, Dallas, Texas; UT Southwestern Medical Center, Dallas, Texas; University of Texas Southwestern Medical Center, Dallas, Texas; UT Southwestern Medical Center, Dallas, Texas; The University of Texas Southwestern Medical School, Plano, Texas; UT Southwestern Medical Center, Dallas, Texas; University of Utah, Salt Lake City, Utah; University of Texas Southwestern Medical Center, Dallas, Texas; UT Southwestern Medical Center, Dallas, Texas; University of Texas Southwestern Medical Center, Dallas, Texas; UT Southwestern Medical Center, Dallas, Texas; The University of Texas Southwestern Medical School, Plano, Texas; UT Southwestern Medical Center, Dallas, Texas; University of Utah, Salt Lake City, Utah; University of Texas Southwestern Medical Center, Dallas, Texas; UT Southwestern Medical Center, Dallas, Texas; University of Texas Southwestern Medical Center, Dallas, Texas; UT Southwestern Medical Center, Dallas, Texas; The University of Texas Southwestern Medical School, Plano, Texas; UT Southwestern Medical Center, Dallas, Texas; University of Utah, Salt Lake City, Utah; University of Texas Southwestern Medical Center, Dallas, Texas; UT Southwestern Medical Center, Dallas, Texas; University of Texas Southwestern Medical Center, Dallas, Texas; UT Southwestern Medical Center, Dallas, Texas; The University of Texas Southwestern Medical School, Plano, Texas; UT Southwestern Medical Center, Dallas, Texas; University of Utah, Salt Lake City, Utah; University of Texas Southwestern Medical Center, Dallas, Texas; UT Southwestern Medical Center, Dallas, Texas; University of Texas Southwestern Medical Center, Dallas, Texas; UT Southwestern Medical Center, Dallas, Texas; The University of Texas Southwestern Medical School, Plano, Texas; UT Southwestern Medical Center, Dallas, Texas

## Abstract

**Introduction:**

Post-operative pain management can be a significant challenge in patients undergoing burn excision. Standard pharmacologic pain management strategies include both opioid and non-opioid medications. Given the national overuse of opioids and the associated negative repercussions, it is prudent that we find ways manage pain with fewer or no opioids. We hypothesize that intraoperative administration of intravenous methadone will reduce the total morphine milligram equivalents (MME) used in the 36 hours following surgery.

**Methods:**

This is a retrospective single-center cohort study of adult burn patients who underwent a first excision of full thickness burn between January 2019 through January 2021. The exposure group received intraoperative methadone while the control group did not. The primary outcome was total MME utilized in the 36 hours following surgery. Secondary outcomes included average pain score and post-anesthesia care unit (PACU) total MME utilized. Chi squared tests were used for statistical analysis of categorical variables and unpaired t-tests were used for continuous variables.

**Results:**

The control group contained 35 subjects and the methadone group contained 19 subjects who did not differ in baseline characteristics. The average burned total body surface area (TBSA) was 9% in the control group and 18% in the methadone group (t(53)=-3.9, p < 0.01). Intraoperative narcotic requirements did differ between groups due to intravenous methadone utilization in the intervention group (t(53)=-15, p < 0.01). In the early post-operative period, the methadone group received 155 MME while the control group received 137 MME (t(53)=-0.36, p = 0.72). While in the PACU, patients in the methadone group received 5 MME while the control group received 12 MME (t(53)=1.60, p=0.12). Patient pain scores did not differ significantly between the groups (t(53)=-0.15, p=0.88).

**Conclusions:**

Intraoperative utilization of methadone for burn surgery did not affect post-operative opioid usage in the first 36 hours or post-operative pain scores in a statistically significant manner. We acknowledge the data set is not large enough to power the study to detect a significant difference; additional data collection is ongoing to include several hundred subjects. Confounding variables such as different multimodal pain regimen techniques, % TBSA, extent of surgical excision, and history of opioid tolerance may exist within this data set. Further analysis is warranted to adequately power this study and account for potential confounding variables.

**Applicability of Research to Practice:**

Intraoperative use of intravenous methadone may reduce use of opioid medications in the early post-operative period.